# Inequities in access to palliative and end-of-life care in the black population in Canada: a scoping review

**DOI:** 10.1186/s12939-024-02173-9

**Published:** 2024-04-25

**Authors:** Nahyeni Bassah, Julia Beranek, Megan Kennedy, Juliet Onabadejo, Anna Santos Salas

**Affiliations:** 1 Faculty of Nursing, College of Health Sciences, University of Alberta, Third Floor Edmonton Clinic Health Academy, 11405 87 Avenue NW, Edmonton, AB T6G 1C9 Canada; 2 Department of Nursing, Faculty of Health Sciences, University of Buea, P.O Box 63, Buea, South West Region Cameroon; 3 Geoffrey & Robyn Sperber Health Sciences Library, Edmonton Clinic Health Academy, 1-150M, 11405 87 Avenue NW, Edmonton, AB T6G 1C9 Canada; 4 BScN Program, School of Health and Wellness, Red Deer Polytechnic, 100 College Blvd, Box 5005, Red Deer, AB Canada

**Keywords:** Inequities, Blacks, Underserved populations, Canada, Palliative and End-of-Life Care, Scoping review, Health equity

## Abstract

**Background:**

Improving equity and early access to palliative care for underserved populations in Canada is a priority. Little is known regarding access to palliative and end-of-life care in the Black population.

**Methods:**

We undertook a scoping review using the framework by Arksey and O’Malley to identify knowledge, access gaps, and experiences of palliative and end-of-life care among Blacks living with life-limiting illnesses in Canada. Primary studies, discussion papers, books, and reports were considered eligible. We followed a comprehensive search strategy developed by an information scientist. Searches were performed in the following bibliographic databases: Medline, EMBASE, PsycINFO via OVID, CINAHL via EBSCOhost, Scopus and Cochrane Library via Wiley. The search strategy was derived from three main concepts: (1) Black people; (2) Canada and Canadian provinces; (3) Palliative, hospice, or end-of-life care. No publication date or language limits were applied. Titles and abstracts were screened for eligibility by one reviewer and full text by two independent reviewers.

**Results:**

The search yielded 233 articles. Nineteen articles were selected for full-text review, and 7 articles met the inclusion criteria. These studies were published between 2010 and 2021, and conducted in the provinces of Ontario and Nova Scotia only. Studies used both quantitative and qualitative methods and included cancer decedents, next of kin, family caregivers and religious leaders. Sample sizes in various studies ranged from 6 − 2,606 participants. Included studies reported a general lack of understanding about palliative and end-of-life care, positive and negative experiences, and limited access to palliative and end-of-life care for Blacks, across all care settings.

**Conclusion:**

Findings suggest limited knowledge of palliative care and inequities in access to palliative and end-of-life care for Blacks living with life-limiting illnesses in 2 Canadian provinces. There is an urgent need for research to inform tailored and culturally acceptable strategies to improve understanding and access to palliative care and end-of-life care among Blacks in Canada.

## Background

There is a growing need to improve equity and early access to palliative care for underserved populations in Canada [1, 2]. Our knowledge is limited regarding access to palliative and end-of-life care for Black populations in Canada. Immigrants and racialized populations, including Blacks, experience significant disparities in access to health care and poorer health outcomes than the wider Canadian population [3, 4]. Approximately 60% of all deaths in Canada result mainly from four life-limiting conditions that require palliative care, namely, cancer, cardiovascular diseases, diabetes and chronic respiratory diseases [5]. Canada’s Black population has an increased risk of developing these life-limiting conditions [6], with Black adults having an increased risk of heart failure and stroke compared to other ethnic groups [4, 7]. Diabetes is 2.1 times more common among Black than among white adults [8]. Black males are among those with high incidence and mortality rates from prostate, liver, and stomach cancers while Black females have a high prevalence of multiple myeloma and breast cancer [6, 9, 10]. These data suggest a growing need for palliative and end-of-life care among Blacks in the coming years. Yet palliative care has been identified as one of the most inequitable areas of health care in Canada [11, 12]. An Ontario study for example, found that cancer patients, who were immigrants from racialized groups had higher rates of aggressive end-of-life care than white immigrants [13].

Canada’s Black population reached 1.5milloin in 2021, which accounts for approximately 4.3% of Canada’s total population [14]. In this article, Blacks refer to people who self-identify as Black and are African, Caribbean, South American, or Canadian [15, 16]. By 2041, the Black population is estimated to rise to about 3.0 million [14]. In 2016, Canadian cities that reported the highest numbers of Blacks were Toronto, Montréal, Ottawa-Gatineau, Edmonton and Calgary. These cities are found in three main provinces: Ontario, Quebec, and Alberta. More than half of Canada’s Black population reported Ontario as their home, with the largest number found in Toronto [16]. Most Black immigrants are first generation and are approaching older ages [16], with nearly 1.3% of the 6.6 million Canadian seniors identifying as Black [5, 16]. This progressively ageing population increases the risk of developing life-limiting conditions such as cancer and therefore highlights the need for palliative care [17, 18].

According to the World Health Organization, palliative care prevents and relieves suffering in people living with life-limiting illnesses, through the early identification, and treatment of physical, psychosocial, or spiritual problems [19]. Evidence suggests that early palliative care improves quality of life and wellbeing for individuals with advanced illness and their significant others [20, 21]. Although significant progress has been made in palliative care in Canada, our understanding of how these services meet the unique needs of Black Canadians living with life-limiting illnesses is limited.

Most Black newcomers in Canada (2011 to 2016) are originally from Haiti, Nigeria, Jamaica, Cameroon and the Democratic Republic of the Congo [16]. Global [22] and regional [23] statistics on palliative care show little awareness of palliative care among the general public and limited availability of palliative care services in these countries. Given this background, it is likely that Black immigrants in Canada have limited knowledge of palliative care. Thus, the purpose of this scoping review was to map the literature on the palliative and end-of-life care knowledge, access gaps, and experiences of the Black Population in Canada.

## Design

The review was guided by the scoping review framework by Arksey and O’Malley [24] and reported in accordance with the PRISMA-ScR [25] and PRISMA-S extensions [26].

A comprehensive systematic search was conducted by an experienced health sciences librarian (MK). Searches were performed in the following bibliographic databases from inception to February 1, 2023: Medline, EMBASE, PsycINFO via OVID, CINAHL via EBSCOhost, Scopus, and Cochrane Library via Wiley. Databases were searched using a combination of natural language keywords and controlled vocabularies, such as MeSH, wherever they were available. The search strategy was derived from three main concepts: (1) Black people; (2) Canada and Canadian provinces; (3) Palliative, hospice, or end-of-life care. In order to increase search sensitivity, no publication date or language limits were applied. See Supplementary file 1 for complete search strategies by database.

Results from the database searches were exported in complete batches and the synthesis review software, Covidence©, was used to deduplicate results and facilitate title/abstract and full-text screening of studies. Titles and abstracts were screened for eligibility by one reviewer (NB) and full text by two independent reviewers (NB and JB). Conflicts were resolved by a third reviewer (AS). Articles, were included in this review if they reported palliative and end-of-life care for Black populations in Canada or included a subgroup analysis of experiences, knowledge and access gaps for this population.

We screened the reference lists of included studies for potentially eligible articles and conducted citation chaining of all included studies through the Scopus database.

Thematic analysis [27] was used to identify and collate codes concerning knowledge, access gaps, and experiences of palliative and end-of-life care among the Black Population in Canada.

## Results

**Fig. 1:**
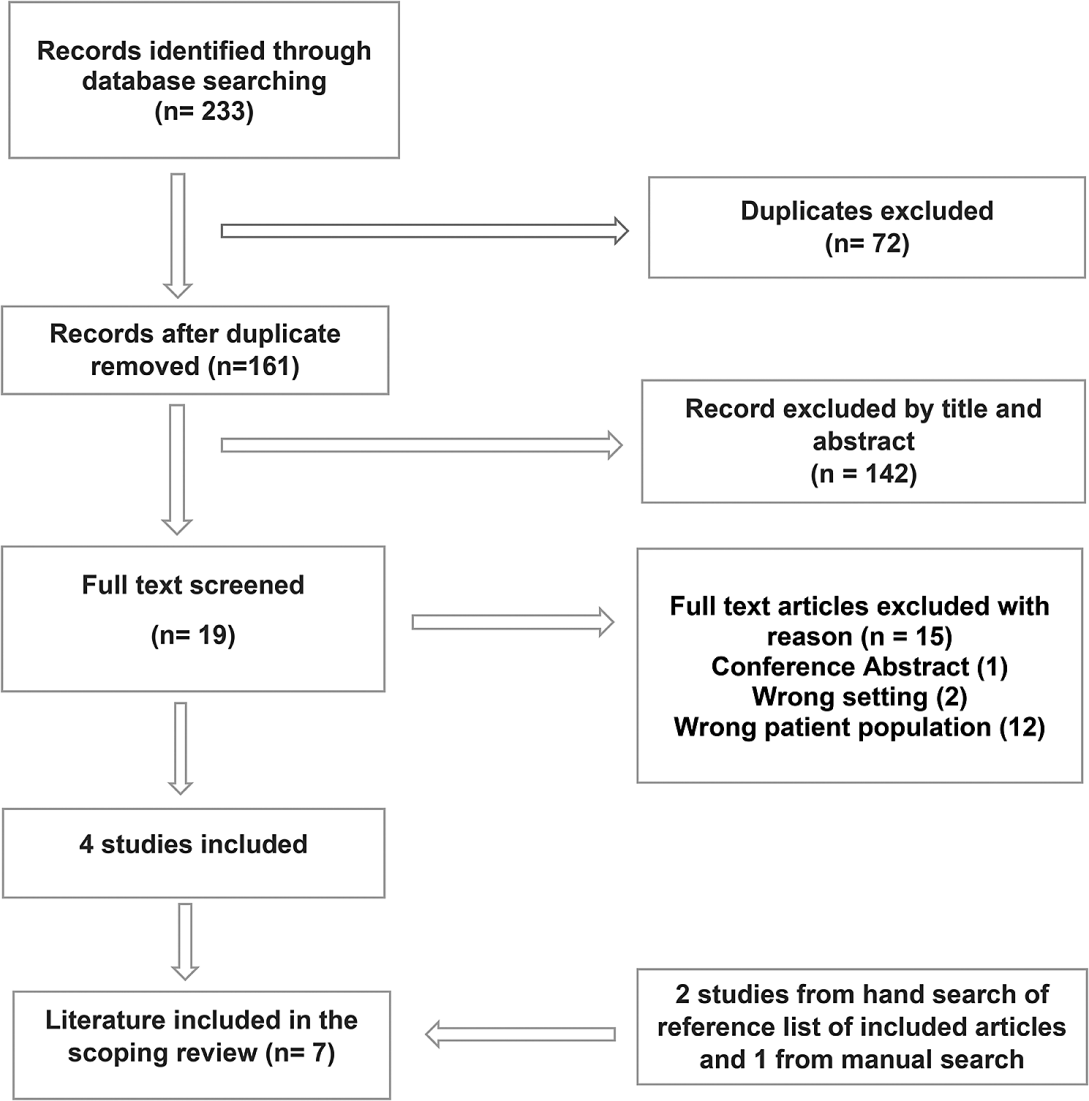
PRISMA flow diagram N:B: the diagram is not displaying properly. Text is covered in some boxes. We can send you a revised figure if that is easier.

The initial database search yielded 233 articles. Of these, 72 duplicates were excluded. Following title and abstract screening, 142 articles were excluded. The full texts of the remaining 19 articles were reviewed and 15 articles were excluded for not meeting the inclusion criteria. Another 2 articles were identified from hand search of reference list of included articles and 1 from manual search. A total of 7 studies met the inclusion criteria and were included for data extraction. The study selection process is illustrated in Fig. 1.

### General characteristics of included articles

Included studies were published between 2010 and 2021. There were four quantitative studies conducted in Ontario [13, 28–30] and three qualitative studies conducted in Nova Scotia [31–33]. The qualitative studies were conducted with Black populations only and sample sizes ranged from 6 to 14 participants. The quantitative studies were with immigrant populations including a proportion of Black participants ranging from 0.3 to 5% of the study population (from 25 to 2606 participants). Studies with immigrant populations mainly reported information about people of African origin, without identifying Blacks from other world regions [28, 29]. Participants in all quantitative studies except for one were cancer decedents, while for all qualitative studies and one quantitative study [28], they were family caregivers, spiritual leaders and/ or next of kin of deceased patients. Three studies reported patient diagnosis and this included cancer [13, 32], and patients living with a late-stage condition [31]. Most Ontario studies used databases of patient records [13, 29, 30], while all Nova scotia studies were conducted in community settings. Study characteristics and key findings are presented on Table 1.

**Table 1:** Overview of included studies on palliative and end of life care for Blacks in Canada

Author and Year	Province	Aim	Population	Design	Key Findings
Chu et al. 2021 [13]	Ontario	To compare end-of-life care between recent immigrants and long-term residents	Cancer decedents including: 13,085 immigrants and 229,471 long-term residents ≥ 18 years at the time of death. Number of Blacks: 1052	Retrospective, population-based cohort study between 2004 and 2015	• Black immigrants have 1.2 times greater risk for aggressive care at end of life than long term residents (OR: 1.20, 95% CI 0.94 to 1.53). • Black immigrants are one of 3 ethnic groups that are least likely to access supportive care (ORs: 0.82, 95% CI 0.72 to 0.93;)
Nayfeh et al. 2021 [27]	Ontario	To measure satisfaction with the quality of inpatient end-of-life care	1,543 next-of-kin of recently deceased patients from various racial backgrounds Number of Blacks: 25	Observational survey	• High level of satisfaction among family members of patients who died in the intensive care unit. Overall Satisfaction score was 8.30(2.09) of 10 • Satisfaction scores among black participants ranged from 8.00–9.00 of 10pionts
Quach et al. 2021 [28]	Ontario	To compare places of care among recent immigrants and long-standing residents in the last 90 days of life.	376 617 deceased individuals, ≥ 18 Number of Africans: 1299	Retrospective cohort study between January 2013, and December 2016.	• Immigrants from Africa used more acute care in the last 90days of life: • Health care service use by immigrants from Africa were as follows: Subacute (6.70%), Acute (58.89%), Community (29.64%), Long-term care (4.77%). • Immigrants from Africa more likely than other participants to receive palliative physician visit in the last 90 days of life [1.35 (1.18–1.53) (*P* <.0001)]
Yarnell et al. 2017 [29]	Ontario	To examine end-of-life care provided to immigrants in the last 6 months of their life.	967 013 decedents who immigrated to Canada between 1985 and 2015. Number from the African region: 2,606	Population-based cohort study from April 1, 2004, to March 31, 2015	• High relative risk of dying in ICU among decedents born in Africa: (95% CI, 1.70-2.00). • Up to 482 (18%) participants of African origin died in the ICU. • High experience of aggressive care at the end of life among recent immigrants from Africa.
Wanda et al. 2014 [32]	Nova Scotia	To examine the role of spirituality at the end of life.	14 participants between 35 to 72 years who were either Caribbean, Canadian Black or African family caregivers or spiritual leaders.	Qualitative In-depth interviews and focus groups	• Role of pastors at end of life is seen as supportive. • More support provided to church members at the end of life, compared to non-church members. • Having faith in God was associated with more peaceful end-of-life experiences than those without faith. • Need for improved healthcare provider awareness of the spiritual needs of African families. • Need for holistic palliative care services: with end of life care plans that include spirituality and involves religious leaders.
Maddalena et al. 2013 [30]	Nova Scotia	To assess knowledge regarding options for palliative and end of life care.	6 African Canadian caregivers of a patient with a late-stage condition who has died within the last 5 years and no sooner than 6 months. Caregivers aged 50-70years	Qualitative: naturalistic inquiry and Participatory Action Research	• Limited knowledge of options for palliative care services. • End of life considered a “family affair.” • Preference for home care and expectation of close family members and community to provide care • Lack of respite and bereavement care. • Financial strain associated with the care of their ill family member • Limited access to information about palliative care services • Need to explore different ways of educating community members about palliative and end of life care services. • Education on available palliative care provided to participants by palliative care team seen as very helpful and hope it continues. • Concern about “strangers” getting in their homes to provide care. • Positive experience with formal healthcare system
Maddalena et al. 2010 [31]	Nova Scotia	Examine the meanings that African Nova Scotians ascribe to their experiences of cancer, family caregiving, and use of complementary alternative medicine (CAM) at end of life.	7 African Canadian caregivers of someone who has died from cancer within the last 3 years and no sooner than 6 months. Three case studies examined: Two African Nova Scotian families and one immigrant family from the Caribbean. Three primary caregivers, and four secondary caregivers.	Qualitative: Case study with in-depth interviews	• Expectation that family members (primarily women) will assume the primary caregiving role in the home for their ill family members with chronic illness or at the end of life. • Reluctance among participants to use conventional institution-based palliative and supportive care. • Considerable hardships experienced while caregiving including financial burden. • Primary caregivers of ill family members often assumed other caregiving roles within the family. • Participants expressed limited knowledge of the supports available within the health system as well as how to access financial supports. • Limited access to bereavement support services. • Importance of spirituality to ill family members at the end of life. • Resignation to fate and “God’s will”. • Home remedies used such as cannabis for pain management, massage and prayer identified as Complimentary and Alternative Medicine. • Use of complementary and alternative medicine due to fear of the health system or denial.

To answer the research question regarding what is known about palliative and end of-life-care for Black populations in Canada, the results of this scoping review are presented in three thematic groupings: palliative and end-of-life care knowledge, experiences of palliative and end-of-life care, and inequities in access to palliative care. These themes are described below.

### Palliative and end-of-life care knowledge

A general lack of understanding about palliative and end-of-life care was identified by only one study [31]. This study reported gaps in knowledge about palliative care services among Blacks in Nova Scotia from the perspectives of family caregivers of deceased patients who were terminally ill. Gaps in knowledge included limited understanding of options for palliative care services, provincially funded palliative care supports, and access to hospice care.

In this study [31], community members described an increased understanding of palliative and end-of-life care services, following a session to educate them about these services. This activity also assisted government stakeholders and health care providers to gain an understanding of the lived experiences of Black family caregivers.

### Palliative and end-of-life care experiences

Studies reported both positive and negative experiences of palliative and end-of-life care among Blacks. Positive experiences included satisfaction with home care and a feeling that health care providers were kind and patient [32], and satisfaction with quality of care in the critical care setting [28]. Negative experiences included concerns about allowing strangers into their homes, not having a consistent home care nurse, and health care providers not meeting their communication and cultural needs [32, 33].

The importance of spirituality at the end of life was highlighted, with views indicating that those with faith in God could have a more peaceful end of life experience than those without faith [31–33]. Participants in these studies reported resignation to fate and the “will of God” and use of prayer when faced with life-limiting illnesses and when experiencing the end of life [32].

End-of-life care was experienced as a family endeavour, with family members having a deep sense of responsibility for assuming primary care of their ill family members in the home. Considerable hardships such as financial strains and fatigue from abandoning jobs to engage as primary caregiver for a sick family member as well as taking care of own family and personal needs, were experienced by family caregivers while giving care [31, 32].

There were also reports of fear of the formal health care system. This was associated with a lack of trust in the health care system emanating from a history of racial discrimination, which resulted in a preference for home care and avoidance of institutionalized care services [32]. There were reports of use of home remedies from herbs, massage, complementary alternative medicine such as cannabis for pain management [32].

### Inequities in access to palliative care

Included studies showed limited access to palliative and end-of-life care for Blacks, across all care settings. This was reflected in reports of increased use of acute care services [29] and aggressive care at the end-of-life [13, 30],, increased relative risk of dying in intensive care [30], and the lowest likelihood of receiving supportive care compared to other populations [13]. Two studies reported underutilization of respite and bereavement services [31, 32] as well as limited use of long-term care facilities [29]. In contrast, data in one study suggested that immigrants of African origin were more likely to receive a palliative physician visit in the last 90 days of life [29].

Familial and religious perspectives on ways to access palliative and end-of-life care were reported. A philosophical view endorsing a family-oriented model of care among Blacks was identified in Nova Scotia. There was a strong preference for services that supported their wish to care for their ill family members in the home setting. There was a strong expectation from the sick family member and the wider Black community that families will assume the role of primary caregivers for their ill family members. Members of the church community received more spiritual and psychosocial support from the church, compared to non-church members [31, 32].

Socioeconomic barriers greatly affected the provision of palliative care in the home as remodelling homes and purchasing assistive equipment such as raised toilet seats and hospital beds put significant financial strain on families. Other reported barriers included the lack of understanding of service options, and lack of culturally and spiritually appropriate services [31–33].

Actionable approaches to improve access and quality of palliative and end-of-life care for Blacks were recommended by participants of the included study, such as: (1) providing information to Black people on end-of-life care expectations and available support services, (2) ensuring consistency of providers in home care [31, 32], (3) ensuring availability of a holistic palliative care service involving religious leaders [33], (4) increasing health care providers’ awareness of the spiritual and cultural needs of Black families [32, 33], and (5) providing more services to support care in the home such as home meal delivery and pharmacy delivery services [32].

## Discussion

Our scoping review findings suggest that research in palliative and end-of-life care focusing on Blacks in Canada is limited. In addition, Black people had a limited understanding of palliative and end-of-life care options and reported mixed experiences.

. In the studies, participants reported positive experiences with palliative care including a feeling that health care providers were kind and patient. They also described negative experiences such as health care providers not meeting their communication and cultural needs. Also reported were concerns about allowing strangers into their homes. Participants’ perspectives emphasized a family-oriented model to palliative and end-of-life care, a preference for home care and inclusion of spirituality as an important dimension of end-of-life care.

Our review findings resonate with findings from studies on palliative and end-of-life for Blacks in other western countries [34–39], as they equally point to the lack of awareness about palliative care and the need to increase knowledge and awareness of palliative care among Black peoples in these countries. Action is also needed to increase access to palliative care for Black populations living with life-limiting illnesses. This entails developing or adapting palliative care modalities that are responsive to the specific sociocultural backgrounds and preferences of Black people. Improving trust in the health care system and dismantling anti-Black racism are necessary steps in this direction. The lack of data and research with Black peoples in palliative care is also a call to undertake studies in this area. We discuss these below.

### Increasing knowledge and awareness

Public awareness of the concept of palliative care is one of the key pillars of a public health strategy for palliative care [40]. With a rising global population and changing demographics there will be a rise in the need for palliative care provision in the community and at home, thus, an increasing need for public engagement and education [41]. Public education has the potential to improve palliative care awareness and strengthen communities’ capacity to care for people with palliative and end-of-life care needs [42, 43]. The lack of awareness of palliative care services among Blacks in Canada suggests the need for the development of culturally appropriate community-based educational programs for Blacks. To our knowledge, no educational interventions to improve understanding of palliative care focusing on Black people in Canada have been developed. In the USA, a community oriented educational intervention consisting of a 10-minute video to educate African Americans about palliative and end-of-life care was found to ease their concerns about palliative care and hospice care as well as increase their consideration to use palliative care [44]. In Ontario, Canada, a culturally tailored educational program to improve access to breast and cervical cancer screening for Black women resulted in an increased awareness of cancer susceptibility, awareness of screening guidelines, and screening self-efficacy [45]. Thus, lessons learned from these initiatives could inform development of similar programs to improve access to palliative care for Blacks in Canada.

### Attending to the sociocultural backgrounds of black people

Culture plays a key role in palliative care due to its grounding in the social practices and beliefs of the community [42, 43]. The impact of culture and spirituality on access to palliative and end-of-life care among Blacks is multifaceted [46]. Studies report unique cultural values and spiritual practices among Black patients and their families at the end of life. This includes, among other things, high family involvement in care and end-of-life decision making, strong religious practices and a belief in a higher being who saves and heals, lack of trust in the health care system and a preference for homecare [47, 48]. These specific sociocultural backgrounds and preferences of Black people needs to be incorporated in palliative and end-of-life care services for this population. Moreover, studies suggest that providers from a different sociocultural and spiritual background may tend to misinterpret Black patient’s wishes. An example is the potential for misinterpretation of Black patient’s statements about hope and miracles as a request for aggressive treatment at the end-of-life [48]. Health care professionals’ understanding of these specific spiritual and sociocultural issues could be enhanced through tailored educational programs [46].

### Advancing equity in access to palliative care

Similarly to our review findings, evidence points to disparities in access to palliative care for Blacks with life-limiting illnesses [34, 36]. Thus, sustained and culturally responsive actions are needed to address this growing inequity. These actions will include, among others, interventions to address the financial, organizational, social, and cultural barriers to service use identified in this review, as well as by studies with Black populations in other high income countries [35, 36, 38, 49]. Additional actions to reduce disparities in access to palliative care include building trust in the formal health care system [50, 51], dismantling anti-Black racism [52], and increasing the number of Black clinicians, educators, and researchers in palliative care [53, 54].

### Implications

Our review findings served to identify research developments in palliative and end-of-life care focussed on Black Populations in Canada living with cancer or other life-limiting illnesses. This review also sheds light on potential strategies to increase access to palliative care for Black Canadians. This review will inform the development of palliative care interventions that are culturally safe and acceptable to racialized populations in Canada. It equally raises the need to advance equity and diversity within the research landscape in Canada [55], with increased research training and mentorship for Black scholars and students to undertake palliative care research. Findings from research with this population will contribute to inform the development of evidence-based approaches to improve their access to palliative and end-of-life care.

### Limitations

Available data is from two provinces only and mostly reflecting the views of a few Blacks of African descent. None of the included studies in this review was conducted with patients, who were currently living with a life limiting condition or experiencing the end-of-life. Although family caregivers are essential care partners [56, 57], reports from patients themselves will provide primary evidence of their needs and experiences living and dying with a life-limiting condition in Canada. This limitation reflects challenges in conducting palliative care research [58, 59] as well as in recruiting and retaining seriously ill patients in research [60–62]. Another limitation was the inclusion of Blacks who were mostly of African origin [29–29]. Statistics Canada reports that between 2011 and 2016, Blacks in Canada originated from over 170 different places with more than 200 ethnic or cultural origins, mostly from Africa (45.8%), and the Caribbean and Bermuda (45.7%) [16]. Thus, the findings of this review are less likely to reflect the views of the general Black population in Canada. This limitation suggests the need for increased diversity of Black study participants’ origins in study samples.

## Conclusion

This review highlights a lack of research on palliative and end-of-life care focussed on Blacks in Canada. Findings suggest limited access to palliative care, limited understanding of palliative care, as well as a combination of both positive and negative end-of-life care experiences among Canada’s Black population in Nova Scotia and Ontario. Research to understand the unique needs of Blacks living with life-limiting illnesses, inform the development of culturally appropriate interventions, and reduce inequities in access to palliative care for this population group is required. Increased research funding and training and mentorship for Black trainees and scholars in the area of palliative care will contribute to achieve this goal.

## Data Availability

All data generated or analysed during this study are included in this published article.
